# Immunoglobulins G of Patients with Schizophrenia Protects from Superoxide: Pilot Results

**DOI:** 10.3390/jpm12091449

**Published:** 2022-09-01

**Authors:** Irina A. Mednova, Liudmila P. Smirnova, Alisa R. Vasilieva, Daria V. Kazantseva, Elena V. Epimakhova, Nina M. Krotenko, Arkadiy V. Semke, Svetlana A. Ivanova

**Affiliations:** 1 Mental Health Research Institute, Tomsk National Research Medical Center of the Russian Academy of Sciences, Aleutskaya Str., 4, 634014 Tomsk, Russia; 2 Department of Psychiatry, Addictology and Psychotherapy, Siberian State Medical University, Moskovsky Trakt, 2, 634050 Tomsk, Russia

**Keywords:** schizophrenia, oxidative stress, superoxide dismutase, abzyme, immunoglobulin G

## Abstract

This study aimed to evaluate the superoxide dismutase (SOD) activity of IgG in patients with schizophrenia. After signing informed consent, we included 67 patients with schizophrenia (34 people with acute schizophrenia and 33 individuals were on outpatient treatment in therapeutic remission) and 14 healthy volunteers. IgGs from blood serum were isolated by affinity chromatography. SOD activity of antibodies was determined spectrophotometrically. We have shown for the first time that IgGs from patients with schizophrenia have SOD activity and this activity is an intrinsic property of antibodies. The maximum increase in SOD activity was registered in the group of patients in therapeutic remission compared with acute schizophrenia (*p* = 0.005) and in healthy individuals (*p* = 0.001). Based on the data of inhibitory analysis using a specific SOD inhibitor enzyme, triethylenetetramine (TETA), we can assume that the mechanism of the SOD activity of IgG is similar to the mechanism of classical enzyme catalysis. According to the kinetic analysis, the affinity of the IgGs to the substrate is higher than that of the classical SOD enzyme.

## 1. Introduction

Schizophrenia is a severe, chronic, and progressive disorder of unknown etiology and pathogenesis. There is controversy over the genesis of schizophrenia, but at the heart of schizophrenia is a violation of the processes of neurotransmission. Oxidative stress is an important pathogenetic factor in schizophrenia since neuronal and receptor damage can be associated with either an increase in the oxidative process or a decrease in antioxidant protection or both [1,2]. An imbalance in the antioxidant defense system is observed both in patients in the first episode of schizophrenia who have not previously used antipsychotics and in patients with chronic schizophrenia receiving drug treatment, which indicates the presence of oxidative stress in schizophrenia [3,4,5]. Thus, the development of oxidative stress in these patients may be based on different processes. The so-called primary oxidative stress is based on the activation of neuroinflammation processes against the background of an acute psychotic state, disturbance of neurotransmission processes, and the balance of calcium-dependent processes in the brain. As a result, generalized oxidative stress begins to potentiate further inflammatory processes. In addition, there is secondary oxidative stress that develops in the body of patients in the background of long-term therapy with antipsychotics [6]. At the same time, it should be noted that typical and atypical antipsychotics affect the body’s redox balance differently [6,7,8].

Data on oxidative stress should be interpreted taking into account the state of the antioxidant system of patients. SOD is the first line of defense against reactive oxygen species; data on its activity in the body of patients with schizophrenia are ambiguous. According to a meta-analysis, SOD activity in plasma and polymorphonuclear leukocytes in patients with schizophrenia significantly decreases, but in serum it significantly increases compared with healthy individuals [9]. Flatow et al. (2013) showed that erythrocyte SOD is significantly reduced in acutely relapsed inpatients, first episode psychoses, and stable outpatients [10]. These data were confirmed by other studies [11,12] while other groups have reported increased SOD activity in schizophrenia [13,14,15,16]. Data may vary due to various methodological approaches, as well as the clinical characteristics of patients, concomitant antipsychotic therapy, and different localization of antioxidants. For example, post mortem brain tissue from patients with schizophrenia demonstrated significantly increased SOD levels in the frontal cortex and substantia innominata [17].

Abzymes or catalytically active antibodies are various classes immunoglobulins that can not only to bind an antigen, but also catalyze biochemical reactions. For the first time, in 1946, L. Pauling had an idea about the similarity of antibodies and enzymes, as well as about the mechanisms that determine their specificity [18]. In 1969, B. Jenks suggested that antibodies obtained by immunization with chemically stable analogues of transition states would catalyze these reactions, and their active centers would contain structural components similar to those for enzymes [19]. Catalytically active antibodies are found in healthy people, as well as in patients with autoimmune, neurodegenerative, and infective diseases, and schizophrenia [20,21,22,23,24]. Several studies have shown the ability of antibodies isolated from the blood of humans and other mammals to dismutate reactive oxygen species [25,26,27,28]. IgG in schizophrenia patients has catalase activity that could play a role in protecting organisms from oxidative stress [29]. Previously, we have shown that the serum antibodies of patients with multiple sclerosis have SOD and catalase activity [29].

This study aimed to evaluate for the first time the SOD activity of IgG in patients with schizophrenia.

## 2. Materials and Methods

### 2.1. Participants

This study was conducted in accordance with the Code of Ethics of the World Medical Association (Declaration of Helsinki 1975) and approved (protocol #142, 14 Mart 2021) by the Local Bioethics Committee of the Mental Health Research Institute Tomsk National Research Medical Center, Tomsk, Russian Federation. All participants signed informed consent to participate in the study. We included 67 patients with schizophrenia, according to the International Statistical Classification of Diseases and Related Health Problems, 10th Revision (ICD-10: F20), and those aged between 18 and 55. We did not include individuals with acute infectious, inflammatory, and autoimmune diseases, as well as patients suffering from an alcohol or drug disorder or taking medicines that can affect biochemical or immunological parameters. All patients underwent a psychometric examination using the Positive and Negative Symptom Scale (PANSS) [30].

The patients were divided into subgroups depending on the condition: 34 people with acute schizophrenia were hospitalized; the remaining 33 patients were on outpatient treatment, had no indications for hospitalization, and constituted the remission group. Patients in therapeutic remission received maintenance doses of atypical antipsychotics (most often risperidone, quetiapine, olanzapine). The control group included 14 healthy volunteers.

### 2.2. Blood Sampling

Blood samples from a cubital vein were drawn after an overnight fast into tubes with a clot activator (to isolate the serum) or with EDTA (to isolate erythrocytes). Patients with acute schizophrenia were bled prior to initiation of antipsychotic therapy. Individuals in remission were bled in the morning before taking medication for the current day. To isolate erythrocytes, 2 mL of venous blood was poured into test tubes, diluted with Hanks solution to 10 mL, and centrifuged at 1500 rpm 3 times for 10 min. The washed red blood cells were subjected to hemolysis with distilled water in a ratio of 1:10. To isolate the serum, the blood samples were centrifuged for 30 min at 2000× *g* at 4 °C. The sera were stored at −80 °C until analysis.

### 2.3. Laboratory Methods

To isolate polyclonal IgGs from the sera of patients and healthy donors, affinity chromatography was performed on the Protein G Sepharose column (HiTrap™ Protein G HP, 1 mL) (GE Healthcare Bio-Sciences, Boston, MA, USA) using an ÄKTA pure chromatography system (GE Healthcare Bio-Sciences) as described before. To confirm the homogeneity of the isolated agent, electrophoresis was performed in a gradient (4–15%) polyacrylamide gel and with high-performance gel filtration under “acid shock” conditions (pH 2.4) on a Superdex-200 HR 10/30 column.

SOD activity of IgGs was determined by assessing the degree of inhibition of the reduction of nitroblue tetrazolium (NBT) in diformazan superoxide radicals, which are generated during the oxidation of xanthine (#X0626, Sigma-Aldrich, Burlington, VT, USA) to uric acid in the presence of xanthine oxidase (#X4500, Sigma-Aldrich) on a Cary 60 spectrophotometer (Agilent Technologies, Santa Clara, CA, USA) at a wavelength of 560 nm. The unit of SOD activity (U) was taken as the difference in the amount of reduced diformazan without the participation of SOD, and the amount of diformazan reduced when this reaction was inhibited by SOD per minute in 1 mL of solution per 1 mg of IgG in the sample (μM diformazan/min/mg of IgG) [28]. A detailed description of the methods used has been given previously [27]. The concentration of IgGs was measured using a Varioskan LUX multi-mode reader (Thermo Scientific, Waltham, MA, USA) based on The Core Facility “Medical Genomics”, Tomsk NRMC. To determine the kinetic parameters of SOD activity of IgG, we used different NBT (#74032, Sigma-Aldrich) concentrations (10, 15, 20, 25, 30, 35, and 37.5 μM). The values of apparent Km and Vmax were calculated based on the rate of superoxide dismutation and NBT concentration. Evaluation of the degree of inhibition of the SOD activity of IgGs was performed using the specific SOD inhibitor enzyme triethylenetetramine (TETA) (#90460, Sigma-Aldrich) at concentrations of 1.5, 1.7, 1.85, 1.95, 2, 2.05, 2.2, 2.4, 2.6, and 5 mM. The SOD activity of IgGs without and in the presence of inhibitor was determined. SOD activity of IgGs without inhibitor was taken as 100%.

Total antioxidant capacity (TAC) of blood serum was measured by a spectrophotometric method (Cary 60 spectrophotometer (Agilent Technologies, Santa Clara, CA, USA)) at a wavelength of 517 nm, using 2,2-diphenyl-1-picrylhydrazyl (DPPH, C18H12N5O6, M = 394.33). As a result of the restoration of DPPH by antioxidants in the blood serum, the solution is decolorized and a change in optical density is detected [31,32]. The degree of color decreases in proportion to the concentration of antioxidants in the sample. The reaction was run by adding 20 μL serum to the incubation mixture (1965 µL 50 mM potassium phosphate (pH 7.4), 15 μL DPPH (working concentration 40 mg/mL)). The decrease in optical density (E) was recorded for 5 min, after which ΔE/min was calculated.

SOD activity of blood erythrocytes 50 mM phosphate buffer (pH 7.4) containing 1 mM EDTA and 1% Triton X-100 was induced in the sample (at the rate of 9 mL of buffer per 1 mL of sample). Centrifugation was carried out at 30,000 rpm for 35 min at +4 °C. Then, SOD activity was determined spectrophotometrically in the resulting supernatant [28]. The unit of SOD activity (U) corresponds to the amount of enzyme that catalyzes the conversion of 1 µmol of substrate in 1 min at +25 °C Calculation of enzyme activity was performed per mg of protein [33].

### 2.4. Statistics

Statistical analyses were performed using the SPSS software, version 23(IBM Company, Westchester, NY, USA). The data were tested for the normality of the distribution by the Shapiro–Wilk test. Between-group differences were evaluated using the Mann–Whitney U test for independent samples with non-normal distribution and ANOVA test for independent samples with normal distribution. The categorical variables were analyzed using the chi-squared test. Description statistics were shown as median with 25% and 75% quartiles (Me [Q1; Q3]) or mean and standard deviation (m ± SD). *p*-values less than 0.05 were considered as significant. Kinetic parameters of SOD activity were estimated using the non-linear regression method in the Origin Pro v.8.6 program (www.OriginLab.com, accessed on 1 August 2022) and were presented as linear transformations using a Lineweaver–Burk plot.

## 3. Results

### 3.1. General and Clinical Characteristics of Study Groups

Patients with schizophrenia and healthy persons were age- and sex-matched. Patients in acute schizophrenia and patients in therapeutic remission were comparable in the age of manifestation and duration of the disease (Table 1).

### 3.2. IgG Purification and Analysis

Attribution of the catalytic activity directly to polyclonal IgG requires checking several stringent criteria, such as the use of specific affinity sorbents for the isolation of antibodies, homogeneity of antibodies obtained by electrophoretic analysis with gradient polyacrylamide gel, and retention of catalytic activity after high-performance gel filtration chromatography under pH shock conditions [34].

**Figure 1:**
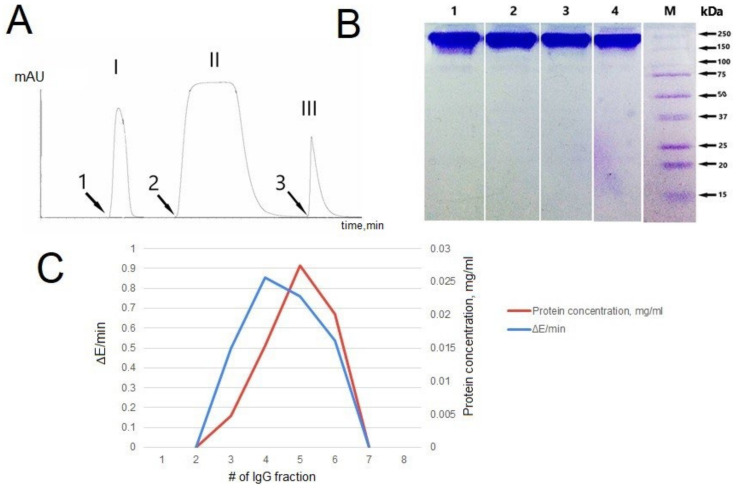
IgG purification and Application of Strict Criteria. (**A**) Profile affinity chromatography of IgGs isolated from the blood serum. 1—application of blood serum; 2—elution with buffer containing 1% Triton X-100 and 0.3 M NaCl; 3—elution with glycine–HCl buffer, pH 2.6. Peak I—proteins with no affinity for the sorbent; Peak II—lipids and non-specifically bound proteins; Peak III—immunoglobulin G. (**B**) Electrophoregram IgGs isolated from the blood serum after Coomassie G250 staining. M—protein molecular weight markers; 1–4—IgGs isolated from the blood serum. (**C**) Protein elution profile of gel filtration of IgGs in pH shock conditions (red line) and the change in the optical density of the solution per minute (∆E/min) in the presence and in the absence of IgGs with SOD activity in the obtained fractions (blue line).

The fulfillment of the first of these criteria is the purification of IgG using specific affinity sorbents. The affinity chromatography profile of IgG from blood serum isolated on a column with immobilized G proteins is shown in Figure 1A. There are three peaks, the last of which corresponds to IgG.

The second criterion is electrophoretic homogeneity of IgG. This was confirmed by the gradient polyacrylamide gel electrophoresis (4–18%) followed by Coomassie G250 staining. One band is shown for the 150 kDa molecular weight band (Figure 1B).

Another important criterion for attributing the catalytic activity of IgGs is the gel filtration of IgG under acidic conditions. Analysis of SOD activity of IgGs from blood serum showed that the activity remained after gel filtration under acid shock only in fractions corresponding to the optical density profile (A280) of antibodies and was not detected in the remaining fractions. Protein profiles of gel filtration of IgGs under shock conditions and elution with 50 mM glycine–HCl (pH 2.6) and 0.3 M NaCl repeated SOD activity profiles of IgGs in the obtained fractions. A graphical comparison of the protein elution profile in gel filtration and the SOD profile of IgG activities in the obtained fractions is shown in Figure 1C. The proof that SOD activity belongs to IgGs is the superimposition of the SOD activity maximum on the top of the chromatographic peak corresponding to IgG.

Based on generally accepted strict criteria for attributing catalytic activity to antibodies, we proved that the studied SOD activity is a property of the IgGs.

### 3.3. Inhibitory Analysis of IgGs’ SOD Activity

**Figure 2:**
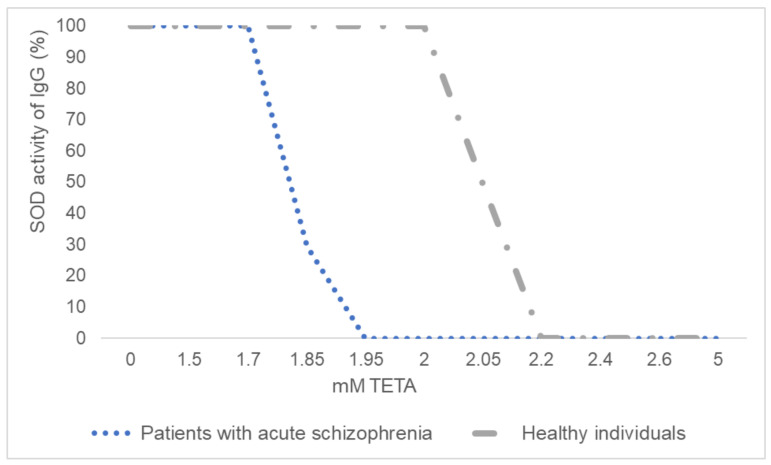
The dependence SOD activity of IgGs on the triethylenetetramine (TETA) concentration. The results are presented as percentages. SOD activity of IgGs without inhibitor is taken as 100%.

The inhibitory analysis was performed on immunoglobulins from schizophrenia patients and healthy individuals. The specific SOD inhibitor TETA suppressed the activity of antibodies in healthy individuals, starting at a concentration of 2.05 mM, and in patients with schizophrenia, starting at a concentration of 1.85 mM. Complete suppression of SOD activity of antibodies in healthy individuals and patients with schizophrenia was found at TETA concentrations of 2 and 2.2 mM, respectively (Figure 2).

### 3.4. Determination of the Kinetic Parameters of IgGs’ SOD Activity

**Figure 3:**
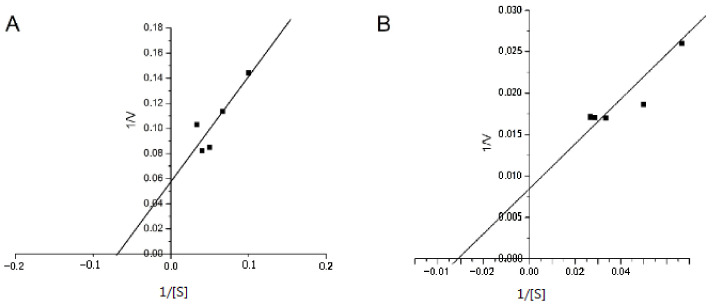
Determination of the kinetic parameters of the IgG SOD reactions using the Lineweaver–Burk plot. (**A**) IgG from healthy person; (**B**) IgG from patient with acute schizophrenia.

We calculated the main kinetic parameters: the Michaelis–Menten constant (Km) and maximum rate (Vmax). Km is numerically equal to the substrate concentration at which the initial rate of the enzymatic reaction is half Vmax. Vmax is the maximum reaction rate observed under conditions where the entire enzyme is in the form of an enzyme–substrate complex [35]. The dependence of the enzymatic reaction rate (V) on the substrate concentration (S) at a constant enzyme concentration is described by a hyperbolic function. To linearize the Michaelis–Menten equation, the Lineweaver and Burk D method of double reciprocals is used. The experimental straight line intersects the abscissa at the point (−1/[S] = 1/Km), and the ordinate at the point (1/V = 1/Vmax). The slope angle tangent is Km/Vmax. We have estimated the Km and Vmax values for the superoxide dismutation rate on the NBT concentration of IgG preparations from a healthy person and patient with acute schizophrenia. The dependence of the superoxide dismutation rate on the NBT concentration in the inverse coordinates is presented in Figure 3.

The analysis showed that Vmax for a patient with schizophrenia was 118.2 µM/min, and for a healthy individual, 17.54 µM/min. The obtained Km values for the same IgG preparations in the patient and healthy person were 32.26 and 14.71 μM, respectively. These data indicated their higher substrate affinity compared to the classical SOD enzyme (Km: 360 μM [35]).

### 3.5. SOD Activity of IgG in Study Groups

**Figure 4:**
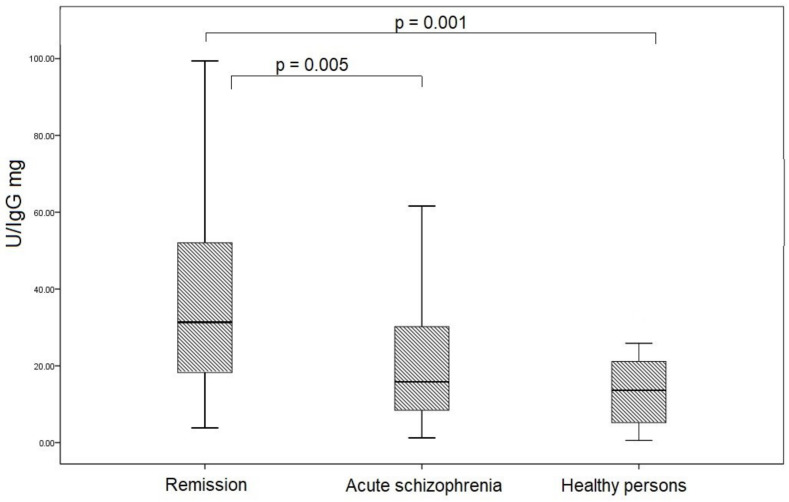
SOD activity of IgG in patients with acute schizophrenia, remission, and healthy persons. Note: To calculate the specific activity, the measured amount of diformazan was used in terms of IgG mg. U corresponds to the amount of enzyme that catalyzes the conversion of 1 µmol of substrate in 1 min at +25 °C.

Superoxide dismutase activity of IgGs was determined by assessing the degree of inhibition of the reduction of nitroblue tetrazolium in diformazan superoxide radicals, which are generated during the oxidation of xanthine to uric acid in the presence of xanthine oxidase. Thus, by determining the amount of diformazan in a sample, it is possible to estimate the activity of SOD IgGs in μM diformazan/min/mg of protein. SOD activity of IgG in patients with schizophrenia was higher than in healthy individuals (*p* = 0.015). The maximum increase in SOD activity was registered in the group of patients in remission (31.35 (18.27; 52.05) µM diformazan/min/IgG mg) compared with acute schizophrenia (15.85 (8.45; 30.20) µM diformazan/min/IgG mg, *p* = 0.005) and healthy persons (13.64 (5.21; 21.14) µM diformazan/min/IgG mg, *p* = 0.001) (Figure 4).

**Figure 5:**
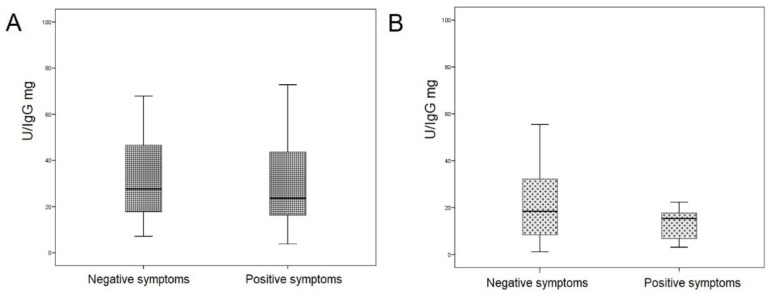
SOD activity of IgG in patients with schizophrenia depending on the leading symptomology. (**A**) Patients with schizophrenia in remission. (**B**) Patients with acute schizophrenia. Note: U corresponds to the amount of enzyme that catalyzes the conversion of 1 µmol of substrate in 1 min at +25 °C.

In the remission schizophrenia group, SOD activity of IgGs was comparable in individuals with leading positive and negative symptoms (23.68 (16.34; 43.59) and 27.71 (17.72; 46.60) µM diformazan/min/IgG mg, respectively; *p* = 0.938; Figure 5A). In the acute schizophrenia group, SOD activity of IgGs also did not differ in individuals with leading positive and negative symptoms (15.41 (6.90; 17.72) and 18.49 (8.48; 32.27) µM diformazan/min/IgG mg, respectively; *p* = 0.401; Figure 5B).

### 3.6. Antioxidant System in Study Groups

**Figure 6:**
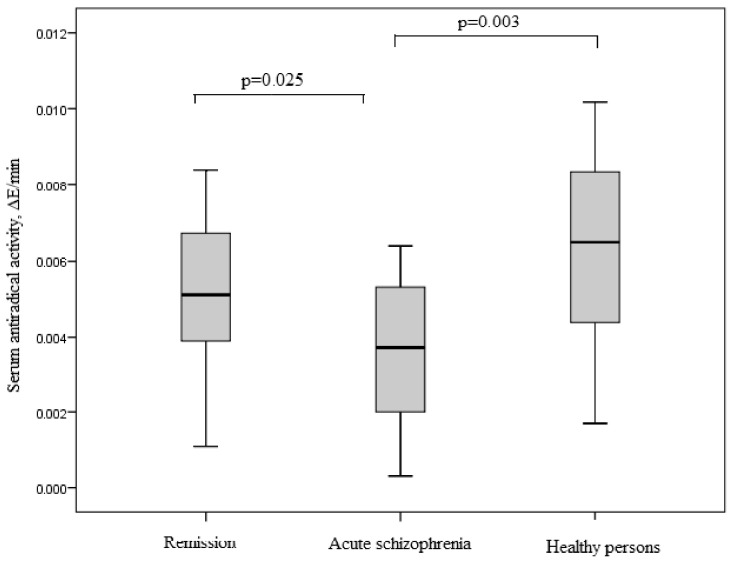
TAC of blood serum in patients with acute schizophrenia, remission, and healthy persons. Note: ∆E/min—change in the optical density of the solution per minute.

As a result of the study, the maximum values of the TAC for healthy people were established. In patients with acute schizophrenia, an almost two-fold decrease in TAC was revealed compared with the control group (*p* = 0.003). Patients in remission showed a significant increase in TAC compared with acute schizophrenia (*p* = 0.025). There were no differences in the values of TAC between the control group and the remission group (*p* = 0.31; Figure 6).

**Figure 7:**
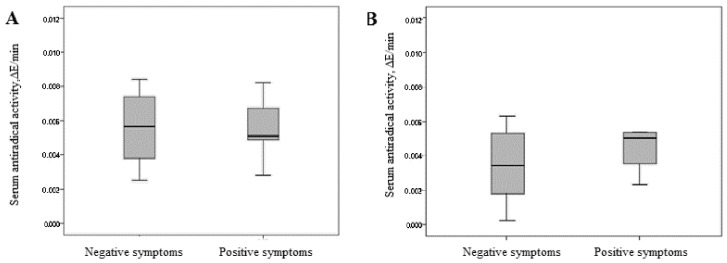
TAC of blood serum in patients with schizophrenia depending on the leading symptomology. (**A**) Patients with schizophrenia in remission. (**B**) Patients with acute schizophrenia. Note: ∆E/min—change in the optical density of the solution per minute.

We found no difference in TAC of blood serum between patients with leading positive and negative symptoms (Figure 7).

**Figure 8:**
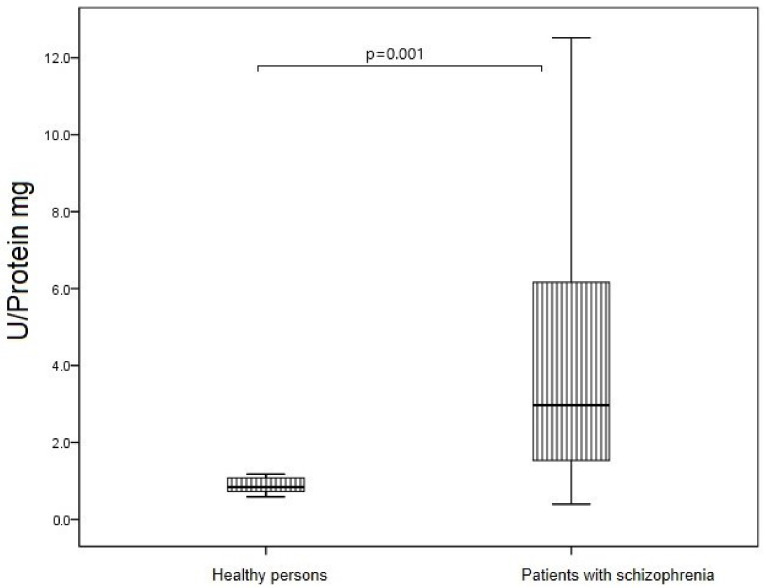
SOD activity of blood erythrocytes in patients with acute schizophrenia and healthy persons. Note: U—corresponds to the amount of enzyme that catalyzes the conversion of 1 µmoof substrate in 1 min at +25 °C.

SOD activity of blood erythrocytes in patients with acute schizophrenia was 4 times higher than in healthy individuals (*p* = 0.001) (Figure 8).

**Figure 9:**
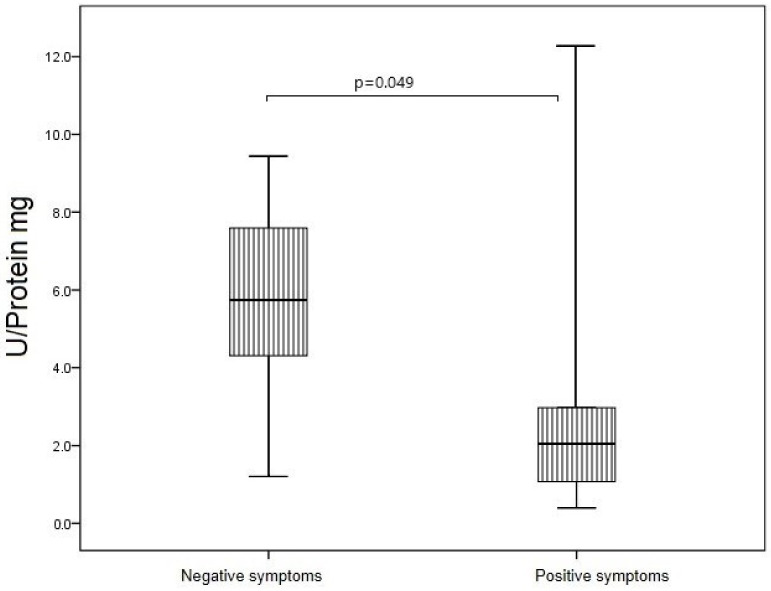
SOD activity of blood erythrocytes in patients with acute schizophrenia depending on the leading symptomology. Note: U corresponds to the amount of enzyme that catalyzes the conversion of 1 µmol of substrate in 1 min at +25 °C.

In the group of patients with schizophrenia, SOD activity of blood erythrocytes was 2.8 times higher in patients with leading negative symptoms than in patients with leading positive symptoms (*p* = 0.049) (Figure 9).

## 4. Discussion

Our work shows for the first time that polyclonal IgG isolated from the blood serum of patients with schizophrenia can catalyze the dismutation reaction of the superoxide anion radical, and this activity is an inherent property of AT, which is confirmed by several strict criteria. Previously, the presence of antibodies with SOD activity was shown in the body of laboratory animals, healthy individuals, and patients with multiple sclerosis [28,36]. In addition, immunoglobulins from healthy people, patients with schizophrenia or multiple sclerosis, and mammals have been demonstrated to exhibit peroxidase and catalase activity [27,28,37]. Nevertheless, the role of catalytic antibodies in the functioning of the immune system in normal and pathological conditions remains poorly understood. Given the high level of oxidative stress in schizophrenia [1,2,3,4,5,9,10,11,12,13,14,15,16,17], we suggested that antibodies with oxidoreductase activity may be involved in protecting the body from the damaging effects of free radicals.

Abzymes can be generated by the formation of antibodies to analogues of the transition states of a chemical reaction or the formation of idiotype–anti-idiotypic antibodies to the active sites of enzymes [38]. Based on the data of inhibitory analysis using a specific TETA inhibitor, we can assume that the mechanism of the SOD activity of IgG is similar to the mechanism of classical enzyme catalysis. It is known that canonical SOD enzymes of mammals are metal-dependent. Three main isoforms of SOD have been isolated in mammals: copper–zinc (Cu,Zn-SOD, SOD1), manganese (Mn-SOD, SOD2), and extracellular (Fe-SOD, SOD3) [39]. In this study, it was found that IgGs with SOD activity are also metal-dependent oxidoreductases, since their activity was suppressed by the TETA inhibitor, a copper ion chelator [40]. At the same time, inhibition of catalytic activity in patients with schizophrenia requires a lower concentration of TETA than in healthy people. Therefore, in terms of functional activity, the studied activity is closest to Cu,Zn-SOD. However, as a result of our work, we cannot yet draw unambiguous conclusions regarding the mechanisms of catalysis characteristic of IgG. The presence of metal ions associated with mammalian immunoglobulin molecules was shown by double jet plasma atomic emission spectroscopy [27]. The ability of immunoglobulins to adsorb various metals on their surface may explain their redox properties. Previous studies also indicate the metal dependence of antibodies with superoxide dismutase activity in healthy individuals and patients with multiple sclerosis [29].

According to the kinetic analysis, the affinity of the abzyme to the substrate is higher than that of the classical SOD enzyme [35]. As a result of the interaction, a sufficiently strong abzyme–substrate complex is formed, which is difficult to dissociate. At the same time, in patients with schizophrenia, the affinity of abzymes to the substrate is 2 times less (Km = 32.26 µM) than in healthy people (Km = 14.71 µM) and 27 times less than in patients with multiple sclerosis (Km = 1.2 μM) [29]. Thus far, we cannot unambiguously explain this phenomenon.

SOD activity of IgG patients with schizophrenia was significantly increased compared to healthy individuals. As previously mentioned, schizophrenia is associated with the development of oxidative stress [1,2,3,4,5,9,10,11,12,13,14,15,16,17]. In particular, hyperproduction of the superoxide anion in schizophrenia was revealed, due to an increase in the activity of NADPH-dependent oxidase [41]. In this state, the body’s own antioxidant systems may not be able to cope and auxiliary defense mechanisms are activated. We suggest that abzymes can be considered as natural proteins for the detoxification of reactive oxygen species in addition to the canonical antioxidant enzymes that work predominantly in tissues. Under conditions of oxidative stress, the antioxidant catalytic properties of abzymes can probably increase to compensate for the insufficiency of enzymatic systems.

In our study, the maximum SOD activity of IgG was recorded in outpatients with therapeutic remission. These patients were on long-term antipsychotic therapy which is known to increase oxidative stress [6,7,8,42]. Animal studies have shown that long-term use of haloperidol can lead to a decrease in the activity and expression of antioxidant enzymes in the brain against the background of an increase in markers of lipid peroxidation [43]. Zhang et al. (2012) showed that risperidone or haloperidol treatment significantly decreased the blood SOD levels of schizophrenia patients [44]. Additionally, other researchers noted an increase in the formation of superoxide anion after haloperidol treatment [45]. We assume that in our study abzymes take on the function of protecting the bodies of patients under oxidative stress conditions. At the same time, the absence of statistically significant differences in the SOD activity of IgG between healthy individuals and patients with acute schizophrenia can be explained by the degree of oxidative stress, in which the resources of the antioxidant system are sufficient to utilize free radicals. These data are confirmed by the higher activity of SOD in the erythrocytes of patients with schizophrenia compared to healthy individuals. Similar results on the increase in SOD activity in schizophrenia were noted in a number of other studies [13,14,15,16,46]. This is also supported by data from studies on the TAC of blood serum. The method for assessing TOC by the ability to reduce the stable radical DPPH is widely used in medical and pharmacological practice to determine the total activity of antioxidants [47]. The lowest serum levels of TOC were found in patients with acute schizophrenia compared with healthy individuals and patients in therapeutic remission. This indicates the greatest dysregulation of antioxidant systems in patients with acute schizophrenia. There were no significant differences in TOC between patients with leading negative and leading positive symptoms. This is supported by other similar studies. Virit et al. (2009) reported that the total TOC in patients with schizophrenia was significantly lower compared with the control group. No differences in oxidative parameters were found between subtypes of schizophrenia or between antipsychotic-treated patients [48]. A decrease in oxidative parameters in patients with schizophrenia is also noted in a number of other studies [49,50]. According to our results for patients in therapeutic remission, there is a positive trend, and the median values of the total antiradical activity are comparable with the group of healthy individuals. This is consistent with the data of Al-Chalabi et al. (2009), who found an increase in the level of antioxidant activity after two months of treatment with olanzapine [51].

Several studies have shown that the activity of the SOD enzyme correlates with the severity of positive symptoms of schizophrenia [7,15,16]. In our study, we found a statistically significant decrease in SOD activity of erythrocytes in patients with leading positive symptoms, and a similar trend was observed for SOD activity of IgG.

A limitation of our study is that we did not take into account the patients’ metabolic status and cigarette smoking. It is known that smoking and metabolic disorders, which are often present in patients with schizophrenia, can lead to increased oxidative stress, so future studies of the oxidoreductase activity of abzymes should take these factors into account. Another limitation of our study is the work with polyclonal antibodies. In future studies, it would be interesting to look at IgG subclasses to better understand the oxidoreductase activity.

## 5. Conclusions

In our study, it was shown for the first time that IgGs isolated from the blood serum of patients with schizophrenia can catalyze the dismutation reaction of the superoxide anion radical, and this activity is an intrinsic property of abzymes which is confirmed by several stringent criteria. In the general group of SOD patients with schizophrenia, the activity of abzymes significantly exceeded that of control subjects. However, when analyzing this activity, depending on the clinical features of the disease, it was shown that this increase was due to patients in a state of therapeutic remission. These patients differed from the group of patients in the acute phase of the disease by the long-term and constant use of antipsychotics. In addition, the greatest dysregulation of antioxidant systems was revealed in acute schizophrenia. Kinetic characteristics of dismutation reactions of the superoxide radical by IgGs are less important than the classical enzyme SOD. Therefore, these abzymes have a lower rate of catalysis compared to the enzyme.

Based on our study, we suggest that abzymes with SOD activity may be a component of the body’s antioxidant system in addition to classical antioxidant enzymes. Antibodies can circulate in the blood for a longer time and utilize free radicals, since they are not affected by serum proteases, unlike enzymes. However, further studies are needed to confirm this assumption.

## Figures and Tables

**Table 1:** General and clinical characteristics of patients with schizophrenia and healthy persons.

Parameter	Patients with Schizophrenia		Healthy Persons	*p*-Value
	Acute Schizophrenia	Remission		
Sex (men/women)	18 (52.9%)/16 (47.1%)	17 (42.9%)/16 (48.5%)	6 (42.9%)/8 (57.1%)	0.913
Age, years	40.00 ± 10.97	38.9 ± 8.93	33.7 ± 8.81	0.132
Age of manifestation, years	22 (19; 30)	22 (20; 27)	-	0.802
Duration of schizophrenia, years	15 (12; 18)	15 (11.5; 20)	-	0.545
PANSS total score	97.5 (89; 105.5)	62,1 (51.3; 71)	-	0.030

Data are presented as mean ± SD (for age); *n* (%) (for sex) and median (Q1;Q3) (for others); PANSS—Positive and Negative Symptom Scale.

## Data Availability

The authors can confirm that all relevant data are included in the article.
